# An In Vitro Potency Assay for Monitoring the Immunomodulatory Potential of Stromal Cell-Derived Extracellular Vesicles

**DOI:** 10.3390/ijms18071413

**Published:** 2017-07-01

**Authors:** Karin Pachler, Nina Ketterl, Alexandre Desgeorges, Zsuzsanna A. Dunai, Sandra Laner-Plamberger, Doris Streif, Dirk Strunk, Eva Rohde, Mario Gimona

**Affiliations:** 1 GMP Unit, Spinal Cord Injury and Tissue Regeneration Center Salzburg (SCI-TReCS), Paracelsus Medical University (PMU), Strubergasse 22, 5020 Salzburg, Austria; a.desgeorges@pmu.ac.at (A.D.); zsuzsanna.somogyi@pmu.ac.at (Z.A.D.); doris.streif@pmu.ac.at (D.S.); e.rohde@salk.at (E.R.); mario.gimona@pmu.ac.at (M.G.); 2 Research Program Nanovesicular Therapies, Paracelsus Medical University (PMU), Strubergasse 22, 5020 Salzburg, Austria; 3 Institute of Experimental and Clinical Cell Therapy, Spinal Cord Injury and Tissue Regeneration Center Salzburg (SCI-TReCS), Paracelsus Medical University (PMU), Strubergasse 22, 5020 Salzburg, Austria; dirk.strunk@pmu.ac.at; 4 University Clinic for Blood Group Serology and Transfusion Medicine, Paracelsus Medical University (PMU), Lindhofstrasse 20, 5020 Salzburg, Austria; s.laner-plamberger@salk.at

**Keywords:** extracellular vesicles, exosomes, T-cell proliferation, immune modulation, mesenchymal stromal cells, mesenchymal stem/progenitor cells

## Abstract

The regenerative and immunomodulatory activity of mesenchymal stromal cells (MSCs) is partially mediated by secreted vesicular factors. Extracellular vesicles (EVs) exocytosed by MSCs are gaining increased attention as prospective non-cellular therapeutics for a variety of diseases. However, the lack of suitable in vitro assays to monitor the therapeutic potential of EVs currently restricts their application in clinical studies. We have evaluated a dual in vitro immunomodulation potency assay that reproducibly reports the inhibitory effect of MSCs on induced T-cell proliferation and the alloantigen-driven mixed leukocyte reaction of pooled peripheral blood mononuclear cells in a dose-dependent manner. Phytohemagglutinin-stimulated T-cell proliferation was inhibited by MSC-derived EVs in a dose-dependent manner comparable to MSCs. In contrast, inhibition of alloantigen-driven mixed leukocyte reaction was only observed for MSCs, but not for EVs. Our results support the application of a cell-based in vitro potency assay for reproducibly determining the immunomodulatory potential of EVs. Validation of this assay can help establish reliable release criteria for EVs for future clinical studies.

## 1. Introduction

Due to their regenerative and immunomodulatory potential, ex vivo expanded organ-specific stromal cells (commonly termed mesenchymal stromal cells or mesenchymal stem/progenitor cells (MSCs)) are widely applied in clinical trials for a variety of therapeutic approaches. However, accumulating data suggest that the therapeutic activity of MSCs is at least in part accomplished by secreted vesicular factors. In pre-clinical studies, extracellular vesicles (EVs) released by MSCs have been shown to improve cardiovascular disease [1], kidney injury [2,3] and neurological disorders [4,5]. In a first case report, repetitive administration of MSC-EVs ameliorated the symptoms of a graft-versus-host disease patient [6].

EVs are small double-lipid membrane vesicles released by many cell types. They contain functional mRNA, miRNA, proteins and lipids, whose transfer to recipient cells enables intercellular communication even between distant regions in the body [7,8,9,10]. EVs are composed of different types of vesicles, including exosomes (40–200 nm), which are of endosomal origin, and microvesicles (150–1000 nm), which directly bud from the cell membrane [11,12]. Due to the lack of methods to physically separate exosomes from small microvesicles, we will use the term “EVs” to refer to vesicles in the size range of exosomes.

MSC-EV-based therapeutics would bear several advantages over cell-based therapeutics, like the lack of self-replication, the possibility of sterile filtration and convenient storage conditions. In vitro assays to reproducibly monitor the therapeutic potency of MSC-EVs are thus urgently needed to facilitate their application and testing in clinical studies. With regard to the immunosuppressive potential of MSC-EVs, published in vitro studies show contradictory results [13,14,15,16,17,18]. We have recently established a convenient dual in vitro immunomodulation potency assay, which reproducibly monitors the effect of MSCs on mitogen-induced T-cell proliferation and alloantigen-driven mixed leukocyte reaction (MLR) of pooled peripheral blood mononuclear cells (PBMCs) [19]. The aim of the current study was to assess whether this potency assay is suitable to evaluate the immunomodulatory effect of MSC-derived EVs. Therefore, EVs derived from three different bone marrow (BM)- and umbilical cord (UC)-MSC primary cultures were generated by differential ultracentrifugation and filtration according to standardized good manufacturing practice (GMP)-grade protocols and compared to their parental cells for their in vitro potential to modulate T-cell proliferation.

## 2. Results

### 2.1. Characterization of EVs from BM- and UC-MSCs

The MSC phenotype of parental cells (three BM-MSC and three UC-MSC primary cell cultures) was evaluated by surface marker expression via flow cytometry analyses (Figure S1) and by standard in vitro tests for osteogenic and adipogenic differentiation potential (Figure S2).

**Figure 1:**
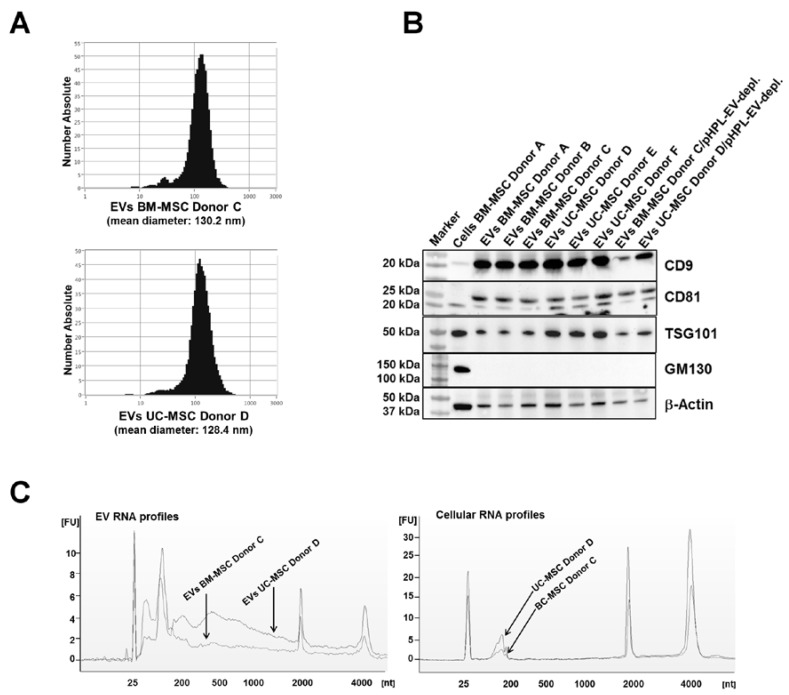
Isolation and characterization of EVs from BM-MSC and UC-MSC cell culture supernatants. (**A**) Size distribution of MSC-derived nanoparticles by nanoparticle tracking analysis (NTA). The mean diameter of EV preparations from three BM-MSC lines was 127.8 ± 2.3 nm; EV preparations from three UC-MSC lines had a mean diameter of 128.5 ± 1.1 nm. Size distributions are displayed for representative nanoparticles from one BM-MSC and one UC-MSC line, respectively. (**B**) Western blot analysis reveals the presence of EV marker proteins CD9 (24 kDa), CD81 (22–26 kDa), TSG101 (45 kDa) and sample processing control β-actin (42 kDa), as well as the absence of EV negative marker GM130 (Golgi protein, 130 kDa) in all EV preparations. Five microliters of EV solutions or 100 µg of BM-MSC lysate as a control were loaded onto the gels. pHPL-EV-depl.: cell culture medium used for EV harvest was depleted of pooled human platelet lysate (pHPL)-derived EVs by ultracentrifugation. (**C**) Detection of vesicular and cellular RNA by Agilent RNA 6000 Pico technique. RNA profiles of EV preparations show enrichment of small RNA species, while cellular RNA profiles suggest the presence of mainly ribosomal RNAs. Profiles of EVs and cells from one BM-MSC and one UC-MSC line are exemplarily depicted (*x*-axis: RNA size in nucleotides; *y*-axis: arbitrary fluorescent intensity).

EVs were isolated from cell culture supernatants by differential centrifugation and filtration (Figure S3). The final volume of EV stock solutions was dependent on the amount of parental cells (EVs from 2 × 10^8^ cells were resuspended in 1 mL Ringer’s lactate solution). The EV preparations contained particles with a mean size of 127.8 ± 2.3 nm (EV preparations from three BM-MSC primary cell cultures) and 128.5 ± 1.1 nm (EV preparations from three UC-MSC primary cell cultures; Figure 1A). The typical EV/exosome-enriched proteins CD9, CD81 and TSG101 were detected in all EV isolates, while Golgi marker protein GM130 was only detected in the control cell lysate (Figure 1B and Figure S4). Compared to cell preparations, the EV preparations were found to be enriched in small RNA species (Figure 1C). Therefore, the preparations fulfilled the minimal requirements of exosomes/EVs [20].

### 2.2. EVs from BM- and UC-MSCs Inhibit Induced T-Cell Proliferation Dose-Dependently

In order to evaluate the immunomodulatory potential of MSC-derived EVs, we determined their effect on mitogen-induced T-cell proliferation. Carboxyfluorescein succinimidyl ester (CFSE) pre-labeled pooled PBMCs were stimulated with the mitogen phytohemagglutinin (PHA) and co-cultured with different ratios of MSCs or EVs for four days. The T-cell proliferation rate was determined by the reduction of CFSE intensity through cell divisions. In all experiments performed, unstimulated pooled PBMCs showed less than 1.65% T-cell proliferation after four days, and PHA-stimulated PBMCs without the addition of MSCs or MSC-EVs exhibited on average of 43.31% T-cell proliferation (Figure S5).

**Figure 2:**
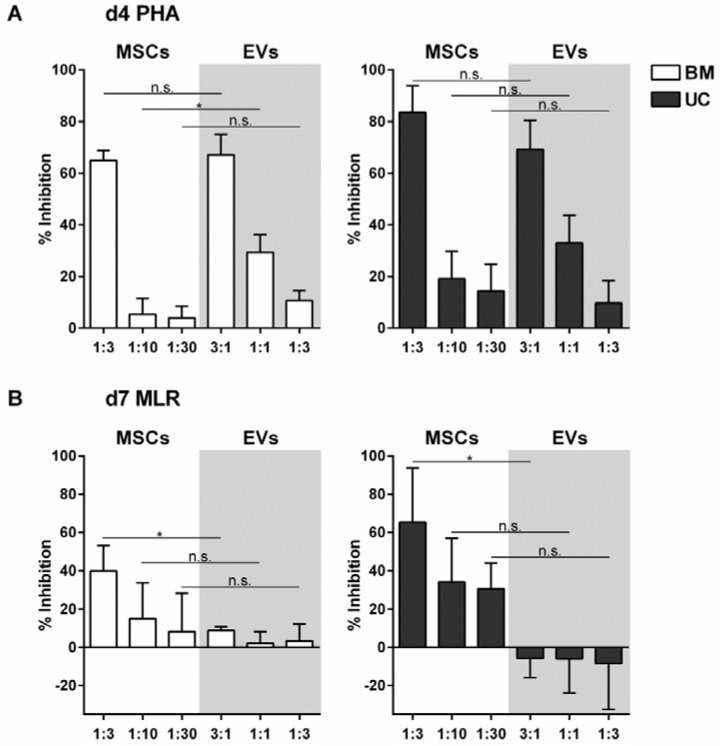
T-cell proliferation assay testing the inhibitory potential of MSCs and their corresponding EVs. Pooled CFSE pre-labeled PBMCs were stimulated with 5 µg/mL PHA (**A**) or via MLR (**B**) and co-cultured with different ratios of MSCs or EVs (grey background) for four or seven days (depicted ratios: cell number MSCs:cell number PBMCs or EVs from cell number MSCs:cell number PBMCs). MSCs and their EVs were either derived from bone marrow (BM, white bars) or umbilical cord (UC, grey bars) samples. Three donors of each group were tested in triplicates. The percentage of successful inhibition of the induced T-cell proliferation is shown (percentage of reduced CFSE-diminishing viable CD3+ T-cells). At Day 4 (d4), EVs prepared from ten-times the amount of parental cells lead to proliferation inhibition comparable to the cells. At Day 7 (d7), the inhibition of the MLR is less effective in all tested groups. The EVs of UC origin show even a stimulation of the T-cell proliferation (negative inhibition) at the analyzed ratios. The mean ± standard deviation (SD) of three independent experiments is shown (n.s.: not significant; * *p* < 0.05).

When PHA-induced PBMCs were cultured in the presence of different amounts of MSC-EVs or MSCs, inhibition of T-cell proliferation was seen in both cases in a dose-dependent manner (Figure 2A and Figure S6A). EVs prepared from ten-times the amount of parental cells exhibited T-cell inhibition comparable to the parental cells (Figure 2A and Figure S6A). EVs derived from BM- and UC-MSCs were equally potent in inhibiting T-cell proliferation. At the cellular level, UC-MSCs had an overall higher capability to repress T-cell proliferation than BM-MSCs (Figure 2A).

### 2.3. MSC-EVs Did Not Inhibit Alloantigen-Driven Mixed Leukocyte Reaction

We next tested, whether MSC-EVs can also inhibit the alloantigen-driven MLR. Unstimulated pooled PBMCs showed negligible T-cell proliferation after four days of culture. However, after seven days, a mean T-cell proliferation rate of 60.58% was observed, indicating MLR in the ten-donor PBMC pool (Figure S5). After a seven-day co-culture of pooled CFSE pre-labeled PBMCs with MSCs, T-cell proliferation was inhibited proportionally to the amount of MSCs added (Figure 2B and Figure S6B). In contrast, the addition of BM-MSC-EVs had only a minor effect on MLR inhibition, and UC-MSC-EVs did not inhibit MLR (Figure 2B and Figure S6B). However, only the maximum amounts of applied MSCs (ratio of MSCs:PBMCs = 1:3) were superior to inhibiting MLR-induced T-cell proliferation of the related MSC-EV preparations (ratio MSC-EVs:PBMCs of 3:1). Lower doses of MSCs did not significantly decrease T-cell proliferation compared to the related MSC-EVs (see Figure 2B).

### 2.4. EVs Released by MSCs under pHPL-EV-Depleted Medium Culture Conditions Inhibit Activation of T-Cell Proliferation Comparably to MSC-EVs Generated in Standard Medium

**Figure 3:**
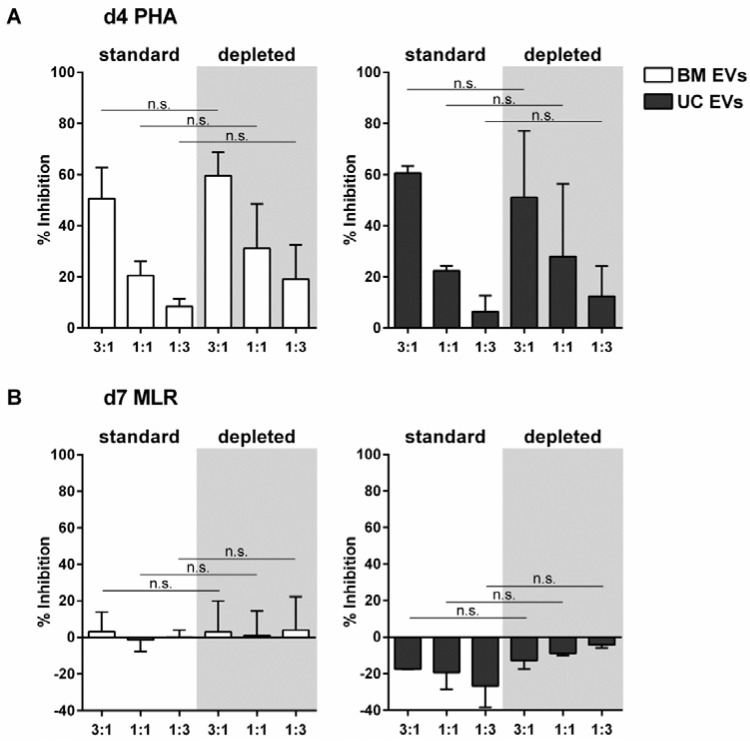
EVs released by MSCs under pHPL-EV-depleted culture conditions still retain the potential to inhibit T-cell proliferation. EVs were derived from BM-MSC donor C or UC-MSC donor D either under standard medium conditions (standard) or under pHPL-EV-depleted medium conditions (depleted, grey background). Pooled CFSE pre-labeled PBMCs were stimulated with 5 µg/mL PHA (**A**) or via MLR (**B**) and co-cultured with different amounts of MSC-EVs in triplicate (depicted ratios: EVs from cell number MSCs:cell number PBMCs). At Day 4 (d4), all EV preparations exhibited inhibition of PHA-induced T-cell proliferation in a dose-dependent manner (A). At Day 7 (d7), BM-MSC-derived EVs had minor MLR inhibitory effects, which were not dose-dependent. UC-MSC-derived EVs had an MLR stimulatory effect, which also was not proportional to the given EV amounts (B). The mean ± SD of preparations tested in triplicate from two experiments is shown (n.s.: not significant).

Culture medium supplements, like fetal bovine serum or human platelet lysate (HPL), contain EVs that can influence the biological behavior of cultured cells [21,22,23], and several of the components co-purify with cell-derived EVs. It is therefore generally recommended to deplete serum supplements of their EVs prior to the production of cell-derived EVs [21,22,24]. We generated EVs from one BM-MSC and one UC-MSC donor cultured in pooled HPL (pHPL)-EV-depleted medium and investigated their potential to inhibit T-cell proliferation. As presented in Figure 3 and Figure S7, MSC-EVs released under pHPL-EV-depleted medium culture conditions have effects on T-cell proliferation comparable to their counterparts derived from standard cell culture conditions. Inhibition of PHA-induced T-cell proliferation by EVs from both organ sources and both culture conditions was dose-dependent (Figure 3A). When co-cultured with pooled PBMCs for seven days, BM-MSC-derived EVs from both culture conditions had minor and variable MLR inhibitory effects (Figure 3B and Figure S7B).

## 3. Discussion

The present study demonstrates that the previously-described potency assay [19] is suitable for evaluating the immunosuppressive potential of MSC-derived EVs based on mitogen-induced T-cell proliferation. At the cellular level, UC-MSCs exhibited a stronger inhibition of T-cell mitogenesis at Day 4 and MLR-induced T-cell proliferation at Day 7 than BM-MSCs. EVs derived from both tissue sources were equally effective at inhibiting T-cell proliferation at Day 4. EVs harvested from pHPL-EV-depleted conditioned medium recapitulated the effect observed with EVs harvested from standard conditioned medium containing pHPL-EVs. The effect of MSC-EVs on MLR-induced T-cell proliferation at Day 7 was generally low; UC-MSC-derived EVs rather stimulated T-cell proliferation at Day 7.

In our assay, EVs prepared from ten-times the amount of cells were required to yield effects comparable to the parental cells. This dependence on high EV doses for in vitro tests may explain why others have found MSC-EVs less effective than the corresponding cells at inhibiting T-cell proliferation [15,16,17]. Adipose tissue (AT)- and BM-MSC-EVs isolated by conventional ultracentrifugation followed by ultracentrifugation on a sucrose cushion failed to suppress T-cell proliferation [17]. However, this purification procedure may have resulted in EV yields too low to monitor an immunomodulatory potential. In two other studies, BM-MSC-EVs were less effective than cells in suppressing T-cell proliferation, but equally effective or even more effective than cells in suppressing B-cell and/or natural killer cell proliferation [15,16]. In another study, which did not include analyses of parental cells, BM-MSC-EVs had no effect on T-cell proliferation, but rather on the T-cell subsets with increased numbers of regulatory T-cells and T helper cells type 2 upon addition of EVs [14]. In contrast, AT-MSC-derived EVs were found to potently inhibit T-cell stimulation [13]. The above studies used MSC-EV preparations derived from various organ sources under different medium conditions, isolated and quantified by different methods, therefore certainly containing EVs of variable amounts in variable compositions.

A major limitation in the EV field is the lack of standardization of current technologies for isolation, quantification and characterization. The current technologies for quantification of EVs are largely inadequate and need to be improved [25]. Total protein measurement appears not useful to quantify EVs from serum-containing conditioned media, ELISA kits still suffer from technical difficulties, NTA and tunable resistive pulse sensing allow quantification and size determination of EVs, but fail to discriminate between protein aggregates and membrane-bounded EVs. For the present study, in which we compare the effect of MSCs with that of MSC-derived EVs, we chose to quantify the EVs based on the number of cells that generate the EVs within a 48-h period (representing a cell-equivalent measurement). Similar approaches have been applied successfully before [6,26].

Under the described in vitro conditions, MSCs added to PBMCs are still able to proliferate and/or secrete anti-inflammatory cytokines in response to cytokines released by T-cells. Thus, MSCs can maintain their effect over the duration of the in vitro test. Yet in vivo, when administered systemically, the majority of MSCs are trapped in the lungs [27,28,29,30], reducing the potential to exert a therapeutic activity or to proliferate. Therefore, the proposed in vitro potency assays may better reflect the in vivo situation of administered non-dividing and non-producing vehicles like EVs than the in vivo condition of administered living cells.

In summary, our data indicate that high doses of EVs may not only be required for in vitro tests, but also for in vivo applications. Improved methods are thus required to manufacture large amounts of EVs following a simple, rapid, reproducible and scalable enrichment process. The potency assay may support the identification of EV preparations with immunomodulatory capacity for future clinical application.

## 4. Materials and Methods

### 4.1. MSC Isolation and Culture

BM aspirates and UC tissues were collected in accordance with the Helsinki Declaration after written informed consent of adult donors. BM-MSCs (*n* = 3) and UC-MSCs (*n* = 3) were isolated as previously described [31,32,33] and tested negative for mycoplasma. After primary cell isolation, the standard cell culture medium was α-modified Minimum Essential Medium Eagle (α-MEM, M4526, Sigma-Aldrich, St. Louis, MO, USA) supplemented with 5 mmol/L N_2_-l-alanyl-l-glutamine (Dipeptiven, 11051014, Fresenius Kabi, Graz, Austria) and 10% pHPL [34,35]. This heparin-free and fibrinogen-depleted standard medium was produced as described earlier [31]. Cells were cultured at 37 °C and 5% CO_2_. MSC immunophenotype characterization by flow cytometry and in vitro osteogenic and adipogenic differentiation potential evaluation were performed as previously reported [21,31].

### 4.2. Preparation of MSC-Derived EVs and Parental Cells

BM-MSCs and UC-MSCs were cultured with standard medium in 4-layered cell factory (CF4) vessels. At 70% (BM-MSCs) and 40% (UC-MSCs) confluence, cells were washed twice with phosphate-buffered saline (PBS), and the culture medium was replaced with fresh standard medium or pHPL-EV-depleted medium (the latter being prepared as described in [21]). After 48 h, EVs were isolated from conditioned media by differential centrifugation and filtration (Figure S3). In brief, cell debris and large vesicles were removed by centrifugation at 2500× *g* for 20 min at 4 °C. The supernatant was ultracentrifuged at 30,000× *g* for 20 min at 18 °C (ultracentrifuge WX-80, fiberlite fixed-angle rotor F37L-8x100, k-factor 168, Thermo Scientific, Vienna, Austria) to remove larger microvesicles. The resulting supernatant was filtered through a 0.22-µm filter and ultracentrifuged at 120,000× *g* for 180 min at 18 °C to pellet EVs. EV-containing pellets were resuspended in PBS and subjected to another round of ultracentrifugation at 120,000× *g* for 180 min at 18 °C. The EV pellets were finally solubilized in Ringer’s lactate solution (0760161/02A, Fresenius Kabi) and sterile-filtered through a 0.22-µm filter. The volumes of Ringer’s lactate solution for EV resuspension was dependent on the parental cell counts and adjusted to yield 1 mL per 2 × 10^8^ parental cells. EV solutions were stored at −80 °C. For T-cell proliferation assays, EV solutions were diluted with assay medium (see Section 4.4.). Parental cells were detached from the CF4 culture vessels by addition of TrypLE Select CTS (A12859-01, Gibco, Denmark). Cell enumeration was performed on cells stained with trypan blue and counted manually using a hemocytometer. MSC immunophenotyping of parental cells was performed by flow cytometry as described [21]. Cell aliquots were stored in liquid nitrogen. Prior to T-cell proliferation assays, thawed cells were cultured for 72–96 h in the appropriate medium (standard medium and/or pHPL-EV-depleted medium).

### 4.3. MSC-EV Characterization

#### 4.3.1. Nanoparticle Tracking Analysis

Particle size and number of EV solutions were determined in a ZetaView Nanoparticle Tracking Analyzer (Particle Metrix, software ZetaView 8-2-31) as described [21].

#### 4.3.2. RNA Isolation and Detection

RNA was isolated from cells cultured in 6-well plates for two days or EV pellets using the mirVANA miRNA isolation Kit (AM1561, Ambion, Austin, TX, USA) according to the manufacturer’s recommendations for total RNA isolation. One microliter of cellular RNA (diluted 1:1000) or vesicular RNA (undiluted) was analyzed with Agilent RNA 6000 Pico chips (5067-1513, Agilent Technologies, Santa Clara, CA, USA) in an Agilent 2100 Bioanalyzer (Agilent Technologies).

#### 4.3.3. Western Blot Analysis

EV solutions (5 µL) were incubated with an equal amount of Laemmli sample buffer (161-0737, Bio-Rad Laboratories, Portland, ME, USA) supplemented with 2-Mercaptoethanol (161-0710, Bio-Rad Laboratories) at 95 °C for 5 min. EV proteins were then separated on 4–15% gradient polyacrylamide gels (456-1084, Bio-Rad Laboratories) and transferred onto nitrocellulose membranes (170-4158, Bio-Rad Laboratories). Precision Plus Protein Dual Color Standard (161-0374, Bio-Rad) served as the protein size marker. Membranes were blocked with 5% non-fat dry milk in Tris-buffered saline containing 0.1% Tween-20 (TBS-T) for one hour at room temperature and probed with primary antibodies against CD9 (sc-13118, Santa Cruz Biotechnology, Dallas, TX, USA), CD81 (sc-7637, Santa Cruz Biotechnology), TSG101 (sc-7964, Santa Cruz Biotechnology) and GM130 (610823, BD Transduction Laboratories, San Diego, CA, USA), all diluted 1:250 in TBS-T containing 0.5% non-fat dry milk, with incubation at room temperature for 4 h. After extensive washing with TBS-T, the secondary antibody (goat anti-mouse HRP-conjugated, K4004, DAKO), diluted 1:200 in TBS-T containing 0.5% non-fat dry milk, was applied for one hour at room temperature. The proteins were detected with ECL Prime Western Blotting Detection Reagent (RPN2232, GE Healthcare, Little Chalfont, UK) and ChemiDoc MP System (Bio-Rad).

After CD9 detection the same nitrocellulose membrane was extensively washed with TBS-T and re-probed with primary antibody against β-Actin, clone AC-74 [36] diluted 1:1000 in TBS-T for four hours at room temperature followed by incubation with secondary antibody and protein detection as described above.

### 4.4. T-Cell Proliferation Assay

The immunomodulatory potential of MSCs and of their corresponding EVs was determined as described previously for MSCs [19]. Briefly, PBMCs from ten randomly selected buffy coats from healthy donors were pooled, stained with CFSE (2188, Sigma) and cryopreserved in liquid nitrogen for further use. Due to the pooling process, a general responder population was generated that enabled stable proliferation rates in all subsequent experiments [19]. The assay medium was RPMI-1640 (R0883, Sigma) supplemented with 10% pHPL, 2 IU/mL preservative-free heparin (L6510, Biochrom, Cambridge, UK), 5 mmol/L N_2_-l-alanyl-l-glutamine (Dipeptiven, 11051014, Fresenius Kabi), 10 mmol/L HEPES (H0887, Sigma), 100 IU/mL penicillin and 100 µg/mL streptomycin (P0781, Sigma). In 96-well flat-bottomed plates (Corning Inc., 734-1796, VWR, Corning, NY, USA), 3 × 10^5^ CFSE pre-labeled PBMCs were seeded per well with different numbers of MSCs (1 × 10^5^, 3 × 10^4^ or 1 × 10^4^ cells) or EVs (EVs from 1 × 10^6^, 3 × 10^5^ or 1 × 10^5^ cells), in a total volume of 250 µL per well. T-cell proliferation was examined either in the presence of 5 µg/mL PHA (L1668, Sigma) after four days or without any additional stimulation after seven days (i.e., stimulated by the MLR due to pooling of ten different PBMC donors). In the case of seven-day culture, 50 µL of assay medium were added per well on Day 4. Proliferation rates were analyzed using a Gallios 10-color flow cytometer and Kaluza G 1.0 software (Beckman Coulter, Inc. Brea, CA, USA). The percentage of viable 7-aminoactinomycin-D-excluding (559925, BD Pharmingen, Mississauga, ON, Canada) and CD3-APC-positive (17-0036-42, eBioscience, San Diego, CA, USA) T-cells was determined for both time points. The time course of the T-cell response and a detailed gating strategy have been previously published [19]. For normalization of all assays, the standard stimulation (PHA only/MLR only) was assigned to a value of 100% (dotted lines in Figures S6 and S7), and the percentage of inhibition was calculated.

### 4.5. Statistical Methods

Values are presented as the mean ± standard deviation (SD). Prism Version 6.00 for Windows (GraphPad Software, La Jolla, CA, USA) was used for statistical analysis. Unpaired *t*-tests were performed. *p*-values < 0.05 were considered significant.

## Supplementary Materials

Supplementary materials can be found at www.mdpi.com/1422-0067/18/7/1413/s1.

## Abbreviations

AT	Adipose tissue
BM	Bone marrow
CF4	4-Layered cell factory
CFSE	Carboxyfluorescein succinimidyl ester
EVs	Extracellular vesicles
GMP	Good manufacturing practice
MLR	Mixed leukocyte reaction
MSCs	Mesenchymal stromal cells; mesenchymal stem/progenitor cells
NTA	Nanoparticle tracking analysis
PBMCs	Peripheral blood mononuclear cells
PBS	Phosphate-buffered saline
PHA	Phytohemagglutinin
pHPL	Pooled human platelet lysate
SD	Standard deviation
UC	Umbilical cord
