# Supplementary material for: An In Vitro Potency Assay for Monitoring the Immunomodulatory Potential of Stromal Cell-Derived Extracellular Vesicles
Source: Int J Mol Sci. 2017 Jul 1;18(7):1413. doi: 10.3390/ijms18071413 (PMC5535905; doi:10.3390/ijms18071413)
Supplement: Supplementary file 1 [file ijms-18-01413-s001.pdf]

Supplementary Materials: An In Vitro Potency Assay for Monitoring the Immunomodulatory Potential of Stromal Cell-Derived Extracellular Vesicles

Karin Pachler, Nina Ketterl, Alexandre Desgeorges, Zsuzsanna A. Dunai, Sandra Laner-Plamberger, Doris Streif, Dirk Strunk, Eva Rohde and Mario Gimona

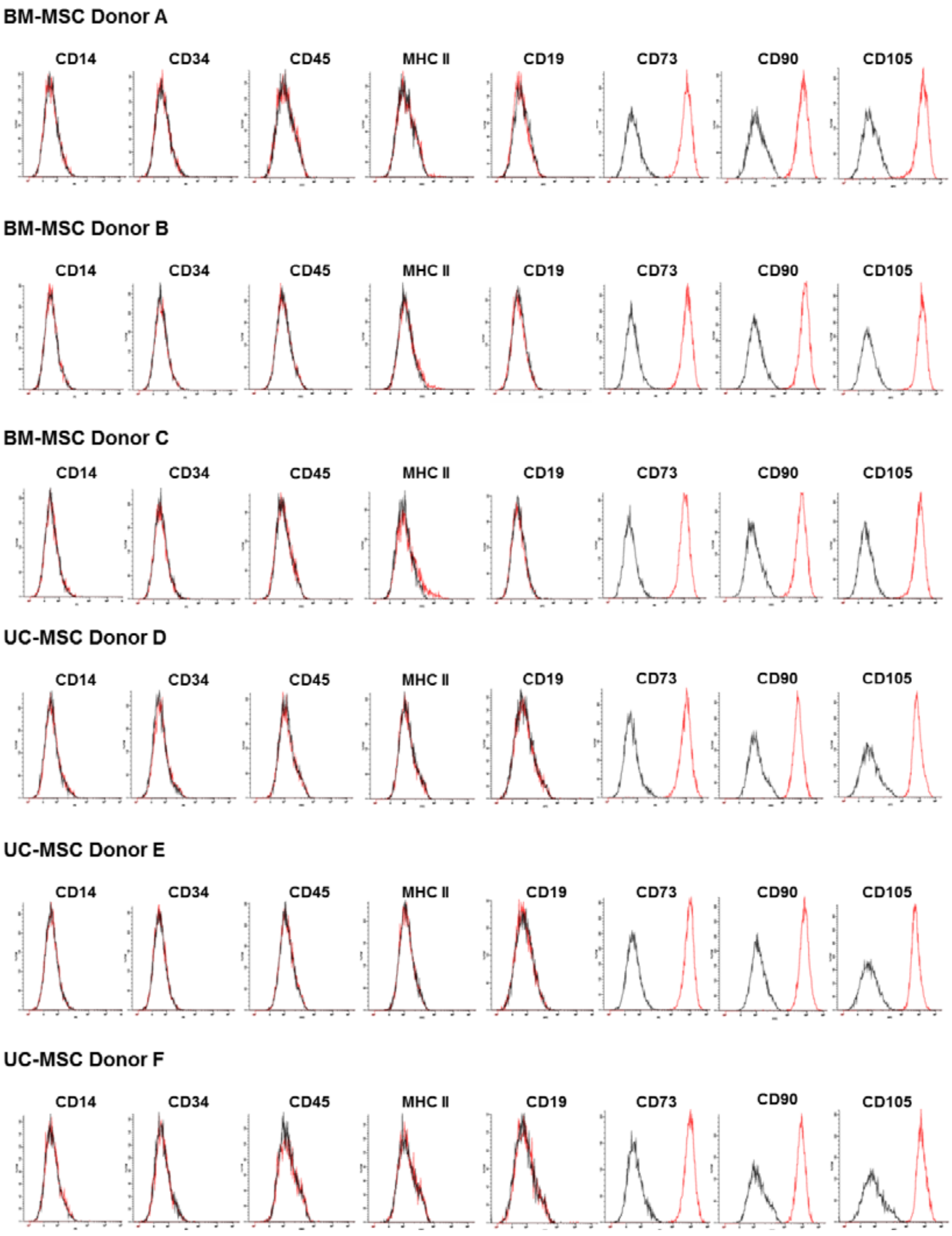

Figure S1. MSC surface marker expression of parental cells. All parental MSC lines were analyzed by flow cytometry using a FACSCanto II flow cytometer (Becton Dickinson), as recently described [21]. Black lines: isotype controls; red lines: specific antibodies.

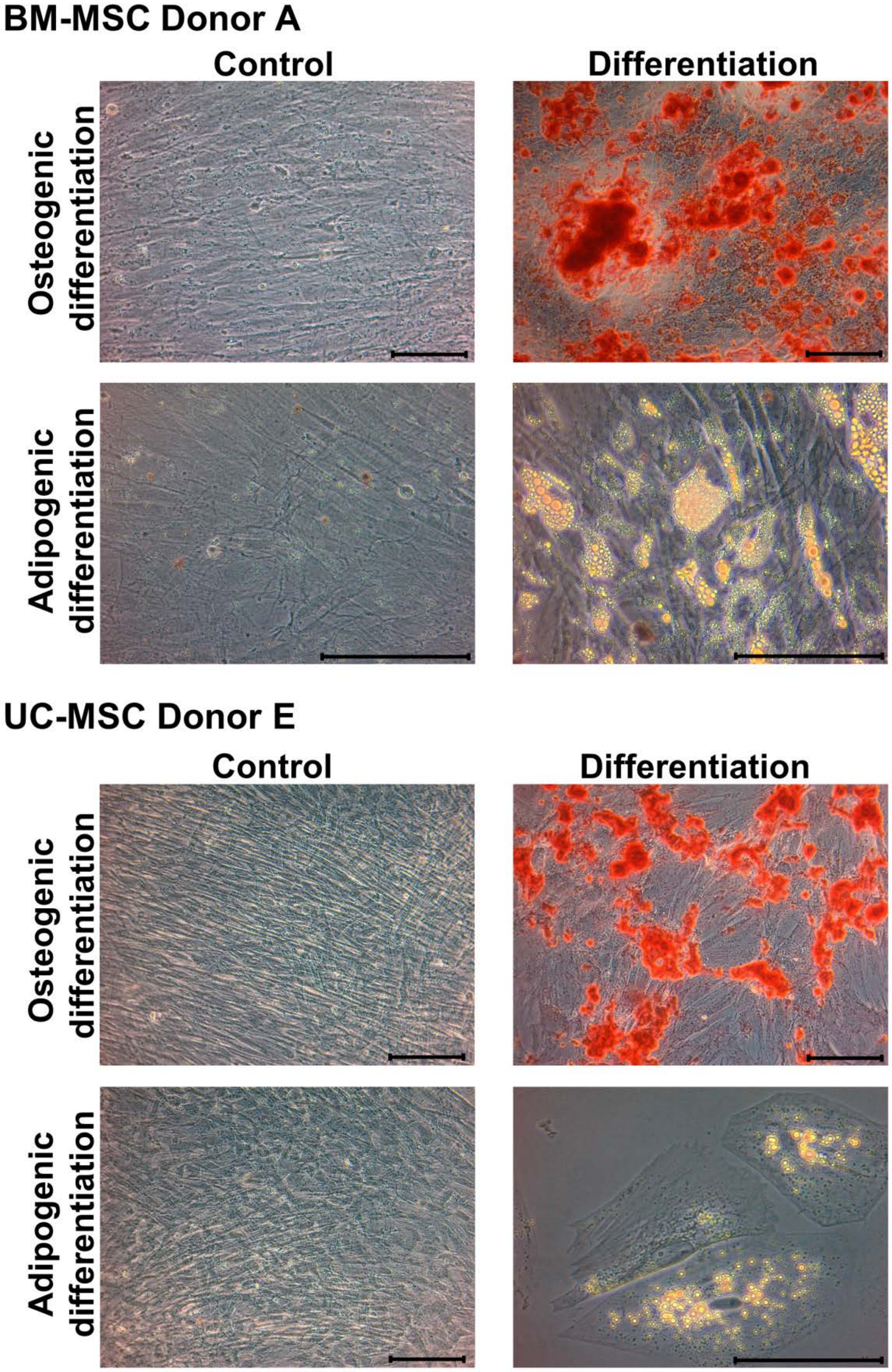

Figure S2. In vitro osteogenic and adipogenic differentiation potential of parental MSCs. All cells were tested for their in vitro osteogenic (Alizarin red staining) and adipogenic (Sudan III staining) differentiation potential as previously described [21,29]. Scale bars: 100 µm. Representative images from one BM-MSC and one UC-MSC donor are depicted.

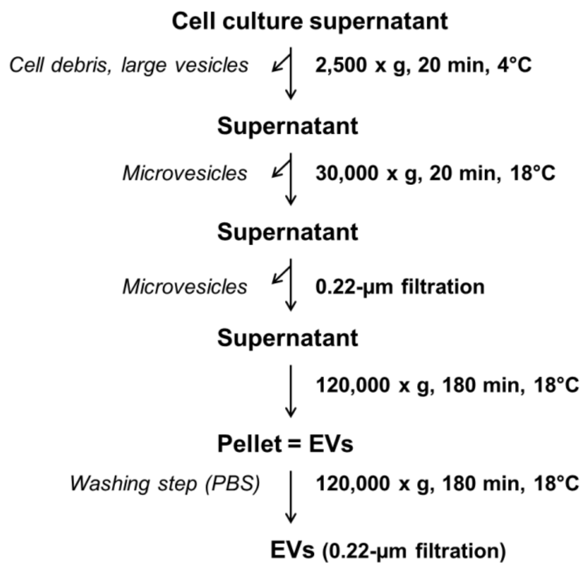

Figure S3. EV enrichment procedure by differential centrifugation and filtration, as described in the Materials and Methods section.

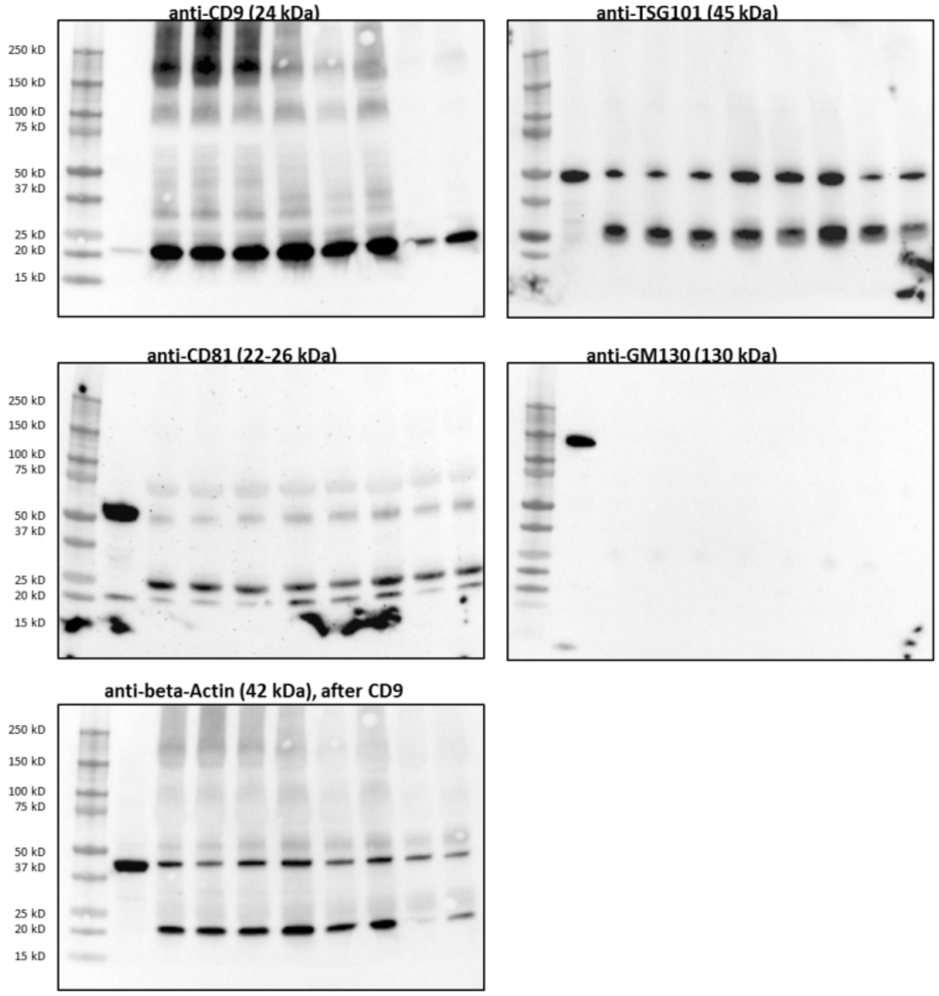

Figure S4. Unprocessed Western Blot images (see Figure 1B). Protein size marker: Precision Plus Protein Dual Color Standard (161-0374, Bio-Rad).

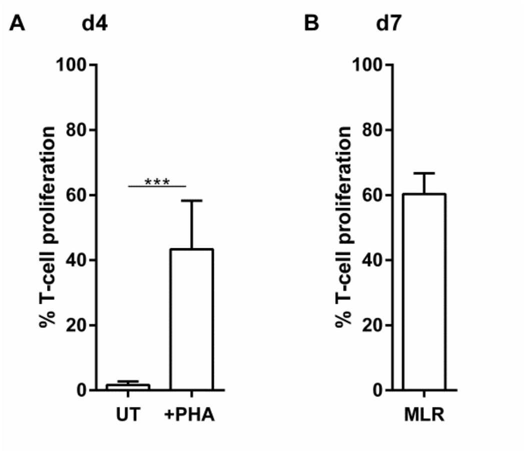

Figure S5. Standard stimulation of T-cell proliferation assays. Unprocessed data showing the T-cell proliferation rate of pooled CFSE pre-labeled PBMCs with or without PHA stimulation at day 4 (**A**) or stimulation via MLR at day 7 (**B**). Mean ± SD of 7 independent experiments is shown (*** $p < 0.001$; UT: untreated).

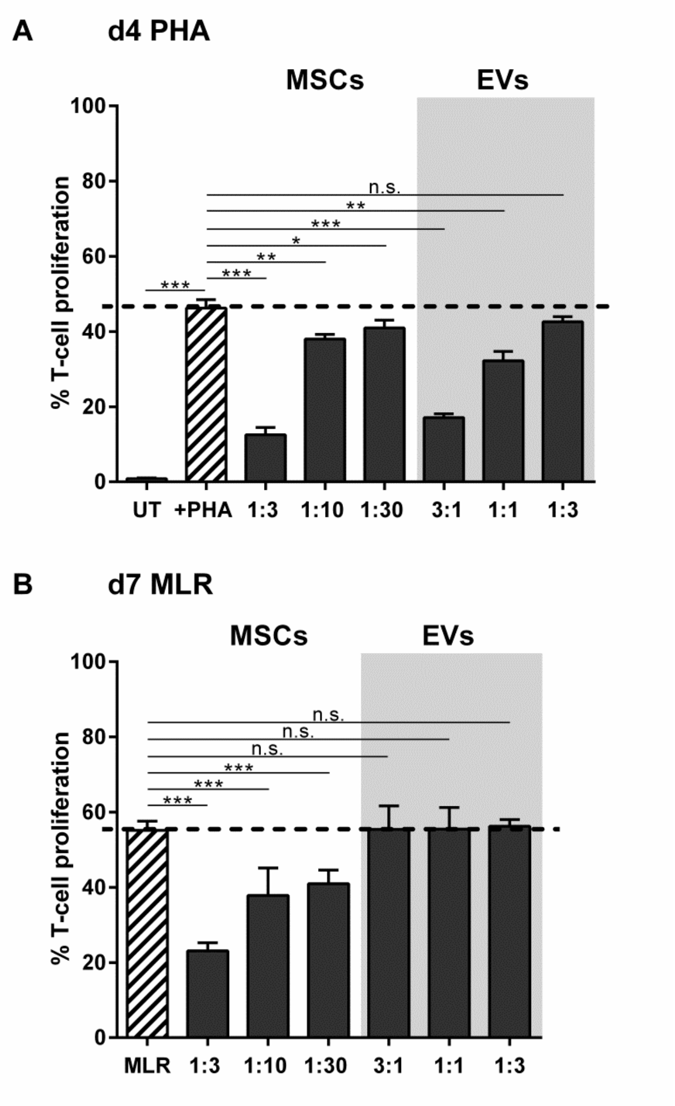

Figure S6. One representative donor of figure 2. Unprocessed data showing the influence on the percentage of T-cell proliferation after administration of UC-MSCs or EVs thereof. Pooled CFSE pre-labeled PBMCs were stimulated with 5 µg/mL PHA (**A**) or via MLR (**B**) and co-cultured with different ratios of MSCs or EVs (grey background) for four or seven days (depicted ratios: cell number MSCs : cell number PBMCs, or EVs from cell number MSCs : cell number PBMCs). At day 4 (d4), MSCs in all tested ratios and EVs in ratios 3:1 and 1:1 significantly inhibit PHA-induced T-cell proliferation. At day seven (d7), only MSCs but not EVs significantly inhibit MLR-mediated T-cell proliferation. Mean ± SD of in triplicates tested preparations from one experiment is shown (n.s.: not significant; * $p < 0.05$; ** $p < 0.01$; *** $p < 0.001$; UT: untreated).

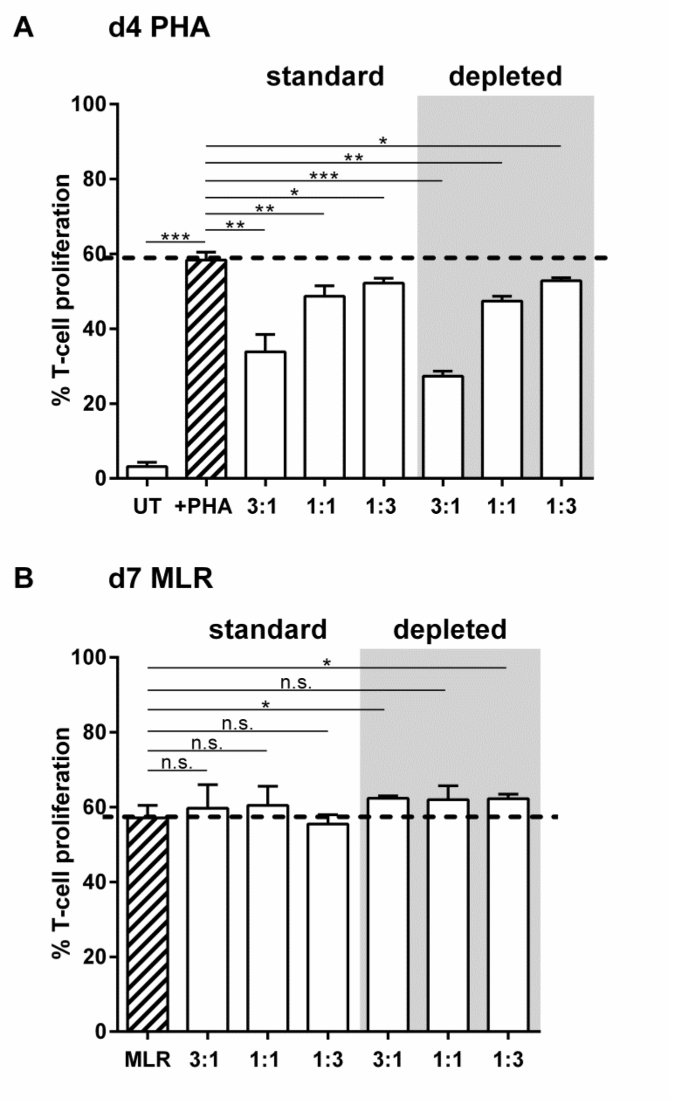

Figure S7. One representative donor of figure 3. Unprocessed data showing the influence on the percentage of T-cell proliferation after administration of EVs derived from BM-MSCs cultured either in standard medium conditions (standard) or pHPL-EV-depleted medium conditions (depleted). Pooled CFSE pre-labeled PBMCs were stimulated with 5 μg/mL PHA (**A**) or via MLR (**B**) and co-cultured with different amounts of MSC-EVs (depicted ratios: EVs from cell number MSCs : cell number PBMCs). At day four (d4), EVs derived from both medium conditions tested in all ratios significantly inhibit PHA-induced T-cell proliferation. At day seven (d7), EVs have either no effect or an MLR stimulatory effect. Mean ± SD of in triplicates tested preparations from one experiment is shown (n.s.: not significant; * $p < 0.05$; ** $p < 0.01$; *** $p < 0.001$; UT: untreated).
